# Naphthoquinones as a Promising Class of Compounds for Facing the Challenge of Parkinson’s Disease

**DOI:** 10.3390/ph16111577

**Published:** 2023-11-08

**Authors:** Thaís Barreto Santos, Leonardo Gomes Cavalieri de Moraes, Paulo Anastácio Furtado Pacheco, Douglas Galdino dos Santos, Rafaella Machado de Assis Cabral Ribeiro, Caroline dos Santos Moreira, David Rodrigues da Rocha

**Affiliations:** 1 Instituto de Química, Universidade Federal Fluminense, Outeiro de São João Batista s/n°, Niterói CEP 24.020-141, RJ, Brazil; thaisbarreto@id.uff.br (T.B.S.); leonardocavalieri@id.uff.br (L.G.C.d.M.); panastacio.fp@gmail.com (P.A.F.P.); dgaldino@id.uff.br (D.G.d.S.); rafaellaribeiro@id.uff.br (R.M.d.A.C.R.); carolmoreira13@gmail.com (C.d.S.M.); 2 Instituto Federal do Rio de Janeiro, Campus Paracambi, Rua Sebastião Lacerda s/n°, Fábrica, Paracambi CEP 26.600-000, RJ, Brazil

**Keywords:** naphthoquinones, Parkinson disease, P2X7 receptor

## Abstract

Parkinson’s disease (PD) is a degenerative disease that affects approximately 6.1 million people and is primarily caused by the loss of dopaminergic neurons. Naphthoquinones have several biological activities explored in the literature, including neuroprotective effects. Therefore, this review shows an overview of naphthoquinones with neuroprotective effects, such as shikonin, plumbagin and vitamin K, that prevented oxidative stress, in addition to multiple mechanisms. Synthetic naphthoquinones with inhibitory activity on the P2X7 receptor were also found, leading to a neuroprotective effect on Neuro-2a cells. It was found that naphthazarin can act as inhibitors of the MAO-B enzyme. Vitamin K and synthetic naphthoquinones hybrids with tryptophan or dopamine showed inhibition of the aggregation of α-synuclein. Synthetic derivatives of juglone and naphthazarin were able to protect Neuro-2a cells against neurodegenerative effects of neurotoxins. In addition, routes for producing synthetic derivatives were also discussed. With the data presented, 1,4-naphthoquinones can be considered as a promising class in the treatment of PD and this review aims to assist the scientific community in the application of these compounds. The derivatives presented can also support further research that explores their structures as synthetic platforms, in addition to helping to understand the interaction of naphthoquinones with biological targets related to PD.

## 1. Introduction

Parkinson’s disease (PD) is a pathology of neurological origin, degenerative of the region present in the central nervous system known as substantia nigra. It was described in 1817, when James Parkinson published his monograph entitled *An Essay on the Shaking Palsy* describing the cardinal symptoms of the disease, which was later named after him [1,2,3].

It is a very common disease, with approximately 8.5 million people affected worldwide in 2019 and, for reasons that are still not fully understood, in the last two decades, the incidence, prevalence and deaths caused by this disease have increased [4,5,6,7].

### 1.1. Epidemiology and Risk Factors

Worldwide, the disease occurs without notable epidemiological differences, the only exceptions being a rapid increase in cases in high-income countries in Europe and a disproportionately rapid increase in new cases in China. The number of PD-related deaths and disabilities has more than doubled in the past two decades [4,5,6,7].

Although PD is age-related, age being the biggest risk factor, where the incidence and prevalence regularly increase with age, it is a mistake to believe that the disease exclusively affects the elderly. For about 25% of affected individuals, the age of onset is less than 65 years and, for 5–10%, it is less than 50 years. For affected individuals younger than 40 years, the term early-onset PD is used [8].

Although PDPAr affects both sexes, women have some differences in relation to men, such as the fact that the incidence of the disease is lower, especially in individuals aged 50 to 59 years, and the age of onset of the disease is higher; however, they have a greater risk of developing dyskinesia (involuntary muscle movements), urinary problems, depression and fluctuations in motor and non-motor response due to their low body weight, leading to a relative overdose [9,10]. Men, on the other hand, live a greater number of years with the disabilities caused by PD and have greater cognitive loss [11]. It is important to emphasize that the health behavior is different between men and women with PD, with late or less frequent access to specialized medical care, which results in undertreatment, including less use of neurosurgical interventions [12,13].

Some environmental factors are linked to the risk of developing PD, such as exposure to certain pesticides and rural life [14]. Likewise, some substances such as 1-methyl-4-phenyl tetrahydropyridine (MPTP) and annonacin can cause nigrostriatal cell death and a form of atypical parkinsonism [15,16,17]. Furthermore, β2 adrenergic receptor antagonists have been associated with an increased risk of PD, whereas β2 adrenergic receptor agonists appear to reduce it [18]. On the other hand, there is an inverse association between the risk of PD and coffee consumption [19], smoking [14], statins [20] and calcium channel blockers [21], while conflicting evidence is available regarding the use of non-steroidal anti-inflammatory drugs [22,23].

Family history is a risk factor for PD and the relative risk in first-degree relatives of PD cases increases by approximately two to three times compared to controls. Familial forms of PD represent 5% to 15% of cases [24].

### 1.2. Pathophysiology

The main pathological characteristics of PD are the loss of dopaminergic neurons with consecutive depigmentation of the substantia nigra and the presence of Lewy bodies. Lewy bodies are round, eosinophilic, intraneuronal inclusions with a hyaline nucleus and a pale peripheral halo that are composed of more than 90 proteins; their main components are α-synuclein and ubiquitin [25]. α-Synuclein has the ability to misfold, become insoluble and form amyloid-rich aggregates in β sheets that accumulate and form intracellular inclusions. Intermediates in this aggregation process are the toxic oligomeric and protofibrillar forms that impair mitochondrial [26], lysosomal and proteasomal function [27], damage biological membranes [28] and the cytoskeleton [29], alter synaptic function and cause neuronal degeneration [30]. At the time of diagnosis, it is presumed that up to 60% of dopaminergic neurons have been lost [31].

Therefore, a model of Lewy body formation and α-synuclein deposition was proposed, which starts in the dorsal motor nucleus of the glossopharyngeal and vagus nerves and anterior olfactory nucleus, with progressive spread to the brainstem and, in subsequent phases, to the mesocortex and allocortex, and finally to the neocortex [32]. α-Synuclein tends to spread through neurons in a similar way to a prion, and this transmission mechanism is probably behind the progression of the pathological changes described above. Furthermore, some data suggest that α-synuclein aggregation may start in the gut autonomic plexus and spread rostrally and that this may be influenced by the gut [33] microbiome.

### 1.3. Disease Mechanisms

The gene coding for α-synuclein, SNCA, was the first linked to PD, and A53T, a mutation that gives α-synuclein a greater predisposition to unfold and aggregate, was the first identified pathogenic SNCA mutation. The similar features of all SNCA mutations include early disease onset, rapid progression of motor signs and notable presence of non-motor features, including cognitive impairment [34,35].

Likewise, impairment of mitochondrial function is another important mechanism for the disease and is usually linked to different genes involved in familial forms of PD [36]. The PINK1 and PRKN genes interact in a mitochondrial quality control pathway, their mutations being the main causes of autosomal recessive and early-onset PD, accounting for up to 77% of cases of juvenile PD. When the age of onset is below 20 years and 10–20% of early-onset PD [37,38,39], however, its progression is slower and responds well to treatment. The DJ-1 gene, on the other hand, plays an important role in regulating the flow of calcium in the mitochondria, which protects the cell from oxidative stress produced by the regulatory activity of dopaminergic neurons and from dopamine toxicity [40]. There is information of mitochondrial DNA mutations in the substantia nigra of PD brains [41].

Likewise, a growing body of evidence links PD to dysfunction in cellular clearance pathways and various genes linked to autophagy [42]. Mutations in the LRRK2 gene affect autophagy and delay the degradation of α-synuclein, which favors its accumulation. These mutations are seen at lower rates in sporadic cases and represent 3–41% of familial PD cases [34,35,43,44].

Likewise, mutations in GBA1, which encodes the enzyme glucocerebrosidase (GCase), are highly prevalent in individuals with PD and currently constitute the most important genetic risk factor, where 5–25% of patients have GBA1 mutations. GBA-linked PD has an early onset and a severe course, particularly with rapid cognitive decline. The GBA contribution is complex and there are interactions with different pathways implicated in the pathogenesis of PD, such as a reciprocal relationship with α-synuclein accumulation, endoplasmic reticulum stress and mitochondrial dysfunction [45,46].

Recently, rare variants of LRP10, a protein that travels between the trans-Golgi network, endosomes and plasma membrane, have been associated with familial PD, dementia from PD and dementia with Lewy bodies. Other proteins included in the same network were linked to PD, including VPS35 and GGA1. However, more research is needed to elucidate the pathogenetic role of alterations in these pathways in PD and other neurodegenerative disorders [47,48]. Why the dopaminergic neurons of the substantia nigra pars compacta are particularly susceptible to degeneration remains unknown, but it has been pointed out that the autonomous pace-making nature of the dopaminergic neurons of the substantia nigra and calcium homeostasis play an important role [49]. Finally, the importance of the microbiome in the pathogenesis of PD has attracted increasing interest and the pathogenetic mechanisms include an alteration in dopamine synthesis and metabolism, dysregulation and inflammation of the immune system and changes in enteral mucosal permeability [50].

### 1.4. Clinical Manifestations

PD presents a set of motor and non-motor characteristics whose expression may vary between individuals; however, all patients must exhibit the main clinical characteristics and respond to dopaminergic therapy to meet the parameters for the diagnosis of PD. Thus, cardinal motor symptoms include tremor, bradykinesia, hypokinesia, akinesia, rigidity, postural instability and gait and speech disorders, among others. Responsiveness of motor symptoms to levodopa administration is an important feature [51].

The clinical picture among patients can be quite heterogeneous, which allows for the definition of different motor subtypes, such as “dominant tremor”, “postural instability and gait difficulty” or “undetermined”. The possible association of the subtype with etiological or prognostic aspects and with the response to treatment is what makes its definition interesting; for example, PD “dominant tremor” has been associated with a slower and less disabling progression compared to “instability posture and gait difficulty” [52,53].

Even though historically defined as a movement disorder, non-motor signs are important aspects of the clinical picture. Non-motor signs range from dysphagia and sialorrhea to autonomic, sensory, sleep, gastrointestinal, cognitive and neuropsychiatric disorders, and are little investigated by physicians; however, they have a great impact on the patient’s health-related quality of life [54,55].

Some other symptoms, generally called prodromal or premotor symptoms, may manifest up to 10 years before diagnosis and the appearance of motor symptoms. The most recognized are constipation, hyposmia, depression and rapid eye movement sleep disorder, but may include anxiety, visual changes and other autonomic disorders. The prodromal phase of PD presents itself as a unique opportunity to identify those at high risk of developing PD before the occurrence of massive neurodegeneration, bringing important information about the mechanisms of the disease and its progression, proving to be a promising therapeutic window for neuroprotective treatments; therefore, many efforts have been made to better recognize this phase [56].

## 2. Quinones as a Privileged Structure for the Development of New Derivatives for PD

**Figure 1:**
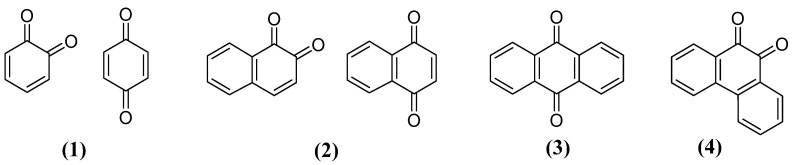
General structures of quinones types.

Quinones are a class of organic compounds widely distributed in nature whose structure has an unsaturated ring of six carbon atoms and two carbonyl groups, which can be situated in an *ortho* or *para* position. Furthermore, quinones are classified due to their aromatic system type as benzoquinones (**1**), naphthoquinones (**2**), anthraquinones (**3**) and phenanthrenequinones (**4**) (Figure 1) [57,58]. In this paper, we will make an overview of natural and synthetic 1,4-naphthoquinones with a neuroprotective effect and potential activity for the treatment of PD.

## 3. Naphthoquinones

Naphthoquinones are the most common type of quinones and compose an important series of natural distribution metabolites of plants, animals, fungi and bacteria [59]. In addition, these substances have great importance in vital biochemical processes [60], and there are many biological effects associated to 1,4-naphthoquinones, the isomer with the highest incidence [61], such as antioxidant/anti-inflammatory [62], antimalarial [63], antitumor [64], tripanocydal [65], antifungal [66] and antibacterial [67]. These effects are related to the formation of reactive oxygen species (ROS), inducing oxidative stress in cells [68], which causes irreversible damage to DNA and proteins leading to apoptosis [69].

**Figure 2:**
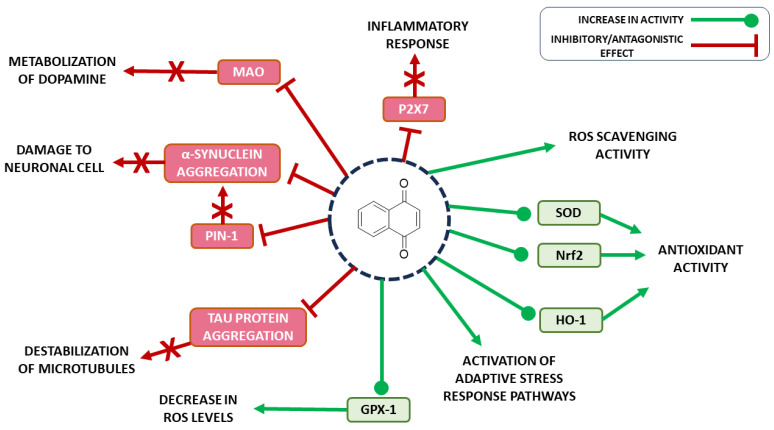
Biological/pharmacological targets of naphthoquinones in the treatment of PD.

However, another important biological effect is the neuroprotective, which occurs through the ability of some 1,4-naphthoquinones to maintain the redox potential of neuronal cells, acting on enzymes responsible for the balance of ROS. This is what protects cells from oxidative stress generated by neurotoxins, capturing free radicals [70]. In addition to the ROS balancing effects, recently, many studies have emerged indicating naphthoquinones as promising molecules in the development of bioactive molecules against PD, exerting their neuroprotective function through different mechanisms (Figure 2).

**Figure 3:**
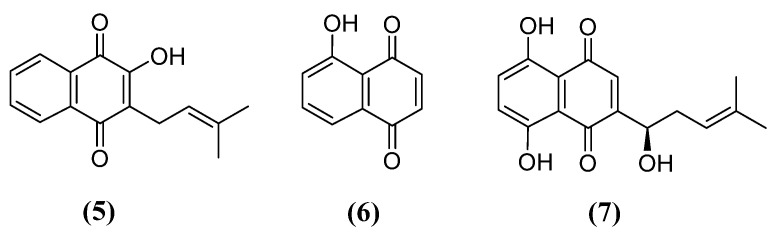
Chemical structures of lapachol (**5**), juglone (**6**) and shikonin (**7**).

In this sense, it is worth emphasizing some 1,4-naphthoquinones related to this neuroprotective effect. First, lapachol (**5**) is isolated from *Tabebuia avellanedae* and extremely used in American folk medicine for the treatment of many diseases, such as cancer, lupulus and infections [71]. Juglone (**6**) is found in some species of the family Jungladaceae—for example, *Juglans regia*, *Juglans nigra* and *Juglans cineraria*—and its use is reported against ringworm, fungal, bacterial and viral infections and as a cure for heat stroke [72]. Finally, shikonin (**7**) is present in the root extract of *Lithospermum erythrorhizon*, named *Zicao*, and was used in traditional Chinese medicine for its anti-inflammatory effects until studies revealed other interesting properties, like anticancer, antimicrobial and wound healing [73] (Figure 3).

### 3.1. Neuroprotective Effect of Naphthoquinones

**Figure 4:**
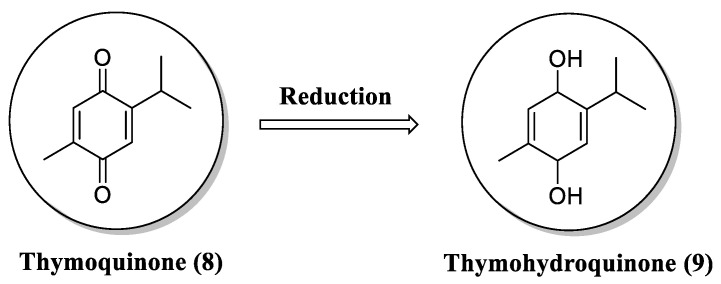
Chemical structure of thymoquinone (**8**) and its metabolite thymohydroquinone (**9**).

Several studies support a neuroprotective effect of naphthoquinones and their derivatives through different mechanisms of action. Natural and synthetic naphthoquinones have been assessed for their antioxidant properties. Oxidative stress is a common pathological feature of traumatic brain injury [74]. Therefore, the use of antioxidant drugs can have a positive impact on the treatment of this neurological condition. It has been identified that the natural naphthoquinone thymoquinone (**8**) and particularly its reduced form, thymohydroquinone (**9**), present strong antioxidant activity (Figure 4) [75]. In this sense, Al-Majed et al. investigated the potential neuroprotective effect of this metabolite, which can be obtained from black cumin (*Nigella sativa*) essential oil, in transient forebrain ischemia-induced neuronal damage in the rat hippocampus [76].

Pretreatment with thymoquinone reduced ischemia-induced neuronal damage by preventing hippocampal neuronal cells death. In addition, thymoquinone also decreased levels of malondialdehyde and increased the levels of antioxidant enzymes in ischemic rats. Thymoquinone and thymohydroquinone avoided in vitro lipid peroxidation in hippocampal homogenate induced by iron-ascorbate with an IC_50_ of 12 and 3 μM, respectively. Thymoquinone has also shown neuroprotective effects on different chemical-induced models. In a model of neuronal injury promoted by chronic exposure to toluene, treatment with thymoquinone (50 mg/kg body weight) for 12 weeks caused a significant morphologic improvement in the neurogenerative status of the frontal cortex in rats [77]. Treatment with thymoquinone also showed beneficial effects on lead-induced brain damage rats [78]. Rats treated with thymoquinone at a dose of 20 mg/kg body weight for one month exhibited similar brain histology to the control group. In an experimental model of arsenic-promoted neurotoxicity, thymoquinone (10 mg/kg body weight) mitigated deleterious effects by suppressing oxidative stress in the cerebral cortex, cerebellum and brain stem [79].

**Figure 5:**
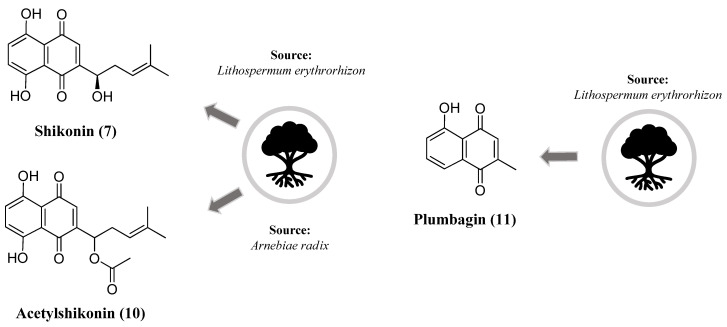
Chemical structure of shikonin (**7**), acetylshikonin (**10**) and plumbagin (**11**).

Based on previous results that showed a neuronal anti-inflammatory role of shikonin (**7**), the main bioactive compound isolated from the roots of *Lithospermum erythrorhizon (*Figure 5), Wang and coworkers studied the potential antioxidant activity of this naphthoquinone in vitro and in a murine cerebral/reperfusion injury. Treatment with shikonin at 50 mg/kg intragastric protected from neurodegeneration after ischemia/reperfusion induction as measured by neurological deficit, cerebral infarct volume and histopathological abnormalities. Shikonin also prevented oxidative stress markers and increased antioxidant defenses in the treated group [80]. Using computational tools and cellular assays, Wang et al. identified among a group of 12 natural compounds that acetylshikonin (**10**) and its derivatives could inhibit the activity of acetylcholinesterase (Figure 5). In addition, these compounds exhibited antiapoptotic activity in vitro by preventing oxidative stress due to a loss of mitochondria membrane potential in mammal neuron cell lines [81]. 

It has been shown that plumbagin (**11**) (Figure 5), a naphthoquinone that can be obtained in the roots of *Plumbago zeylanica* L., exerts neuroprotective effects by modulating multiple essential pathways. Upon oxidative stress signals, the nuclear factor E2-related factor 2 (Nrf2) is activated and binds to the antioxidant response element (ARE), upregulating the expression of genes coding for antioxidant defense mechanisms [82]. The incubation of the human neuroblastoma cell line with plumbagin increased the Nrf2 nuclear localization and expression of Nrf2/ARE-dependent genes. Plumbagin also protected against oxidative-stress-induced cell death in the neuroblastoma cell line and rat primary cortical neurons after oxidative and metabolic stimulus. This protective effect was reversed by RNA interference-mediated Nrf2 silencing. In vivo experiments showed that plumbagin treatment reduced brain damage and neurological deficit after ischemic injury [83]. In an isoflurane-induced neurotoxicity model, plumbagin treatment reduced apoptosis and the expression of pro-apoptotic factors in the hippocampus. Moreover, plumbagin downregulated the PI3K/Akt pathway while upregulating ERK1/2 levels [84]. In the LPS-activated microglial BV-2 cell line, treatment with plumbagin inhibited nitric oxide production more potently than the selective iNOS inhibitor L-N6-(1-iminoethyl) lysine (L-NIL). Also, immunofluorescence assays showed that plumbagin inhibited inducible nitric oxide synthase (iNOS) expression and pro-inflammatory cytokines [85]. 

**Figure 6:**
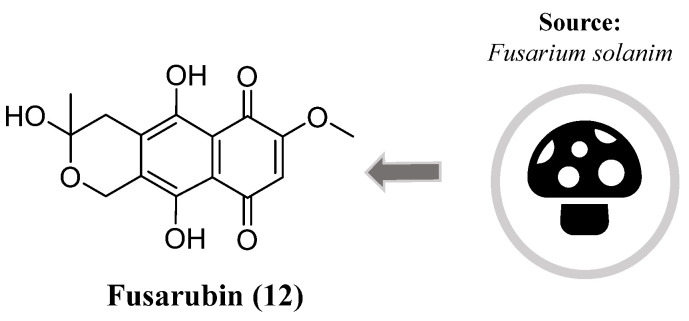
Chemical structure of fusarubin (**12**).

Corroborating the high potential of naphthoquinones as source of effective neuroprotectors, Choi et al. identified the compound fusarubin (**12**), isolated from *Fusarium solanim* (Figure 6), an endophytic fungus present in leaves of the medicinal plant *Morus alba* (Figure 6). This naphthoquinone exhibited reactive oxygen species (ROS) scavenging activity, preventing oxidative-stress-mediated cell death in the murine hippocampal HT22 cell line. Using an in silico approach, the authors proposed that the mechanism of action of this compound may be related to the modulation of ubiquinone levels [86].

**Figure 7:**
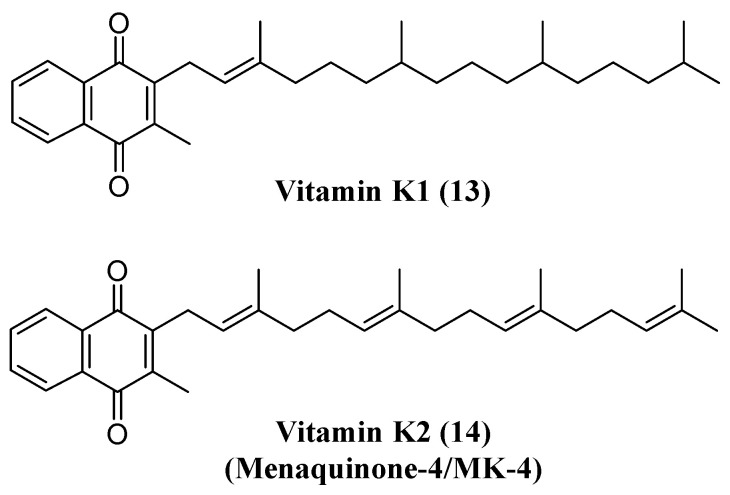
Structure of vitamin K1 (**13**) and K2 (**14**).

Vitamin K (**13**) has also been investigated for neuroprotective actions against different neurological insults. It has been demonstrated that vitamin K, or, more specifically, menaquinone-4 (**14**) (MK-4), is present in brain tissues [87]. In an experimental model of ischemia/reperfusion injury, MK-4 exerted its beneficial effects by inhibiting neuroinflammation markers and neurotoxicity. In addition, MK-4 treatment improved behavioral and cognitive parameters after ischemic insult. MK-4 administration resulted in the elevation of superoxide dismutase (SOD) activity, an essential antioxidant defense mechanism [88]. In agreement with these results, vitamin K prevented cell death mainly by avoiding intracellular glutathione (GSH) depletion in a chemical-induced neurotoxic model with methylmercury [89]. In a different neurotoxicity model induced by amyloid-(Aβ), vitamin K also suppressed the death of neuronal cells, production of ROS and caspase-3 activation, suggesting that a protective effect may occur by Aβ-mediated apoptosis. In this work, it was identified that the vitamin K protection mechanism might be related to the phosphatidylinositol 3-kinase (PI3K) signaling pathway (Figure 7) [90]. 

**Figure 8:**
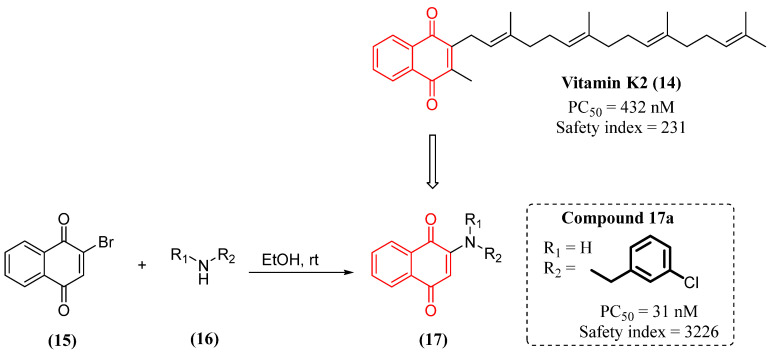
Synthesis and in vitro activity of vitamin K derivatives.

Considering these actions, Josey and collaborators designed a series of vitamin K derivatives and studied the neuroprotective effect of these compounds. They employed a chemical-based approach to modify and optimize the vitamin K pharmacophore to obtain new derivatives with improved pharmacological properties. Particularly, one derivative (**17a**) showed superior protective potency in vitro and a higher safety index with low toxicity (Figure 8) [91].

**Figure 9:**
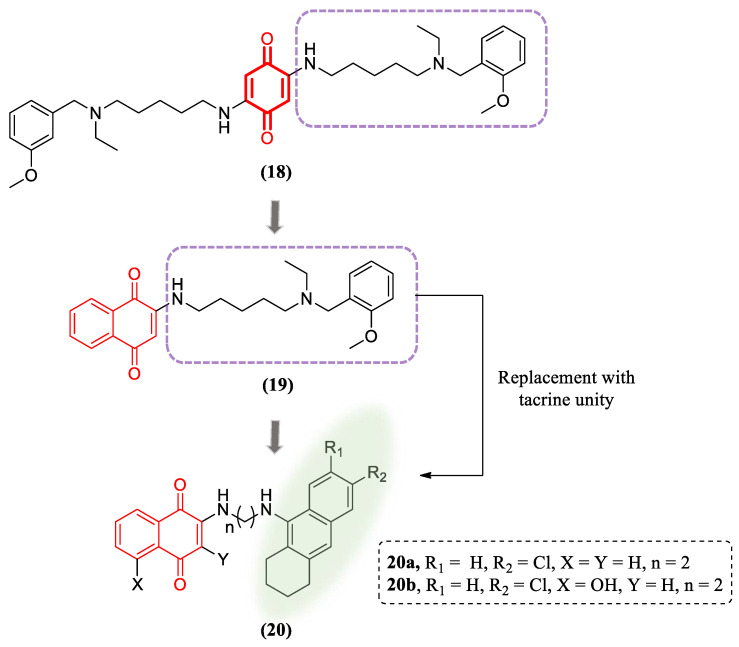
Hybrid compounds containing naphthoquinone and tacrine units.

Other synthetic naphthoquinones have also been investigated with regard to neuroprotective effects. Nepovimova’s group designed and synthetized a group of hybrid molecules containing a naphthoquinone core associated to tacrine, the first drug released for the treatment of Alzheimer disease (Figure 9). The synthetic strategy had as a starting point the compound memoquine (**18**), which exhibited inhibitory activity for acetylcholinesterase (AChE) and amyloid-β aggregation, besides a free-radical scavenging function. Following a structural simplification approach, they identified the compound **1**, which retained the promising biological activity of the parental compound. For the synthesis of the hybrid compounds **20**, they employed a convergent approach and kept the 1,4-naphthoquinone core that was associated to the tacrine moiety through a methylene linker with a properly sized chain. Notably, two compounds exhibited excellent performance in the inhibitory AChE and Aβ aggregation assays. These compounds showed a good safety profile on mouse cortical neurons Neuro2A (N2A) and primary rat cerebellar granule neurons. In addition, the compounds prevented Aβ-induced neurotoxicity and tert-butyl hydroperoxide (TBH)-mediated ROS production [92]. 

**Figure 10:**
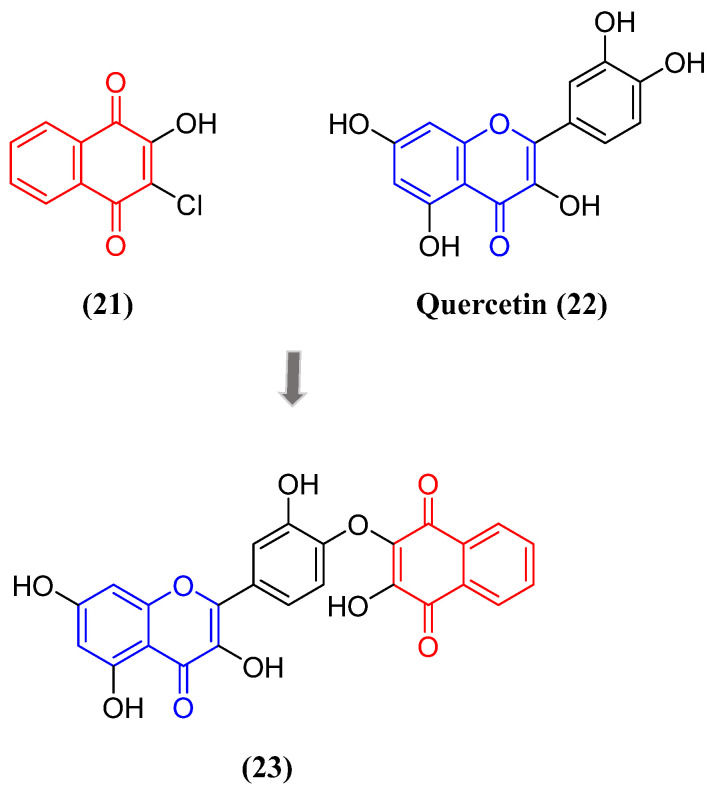
Structure of hybrid compound containing naphthoquinone–quercetin units.

Neurological diseases can be triggered by the persistent activation of microglia and release of pro-inflammatory cytokines. Škandík and collaborators associated two electrophilic pharmacophores to generate a hybrid naphthoquinone-flavonoid derivative and studied its effect on the modulation of the Nrf2 pathway in BV-2 microglial cells (Figure 10). This derivative at a non-toxic concentration reduced iNOS, COX-2 and TNFα levels in LPS-stimulated microglia. In addition, the authors also observed upregulation of antioxidant mechanisms upon treatment with this compound [93]. 

**Figure 11:**
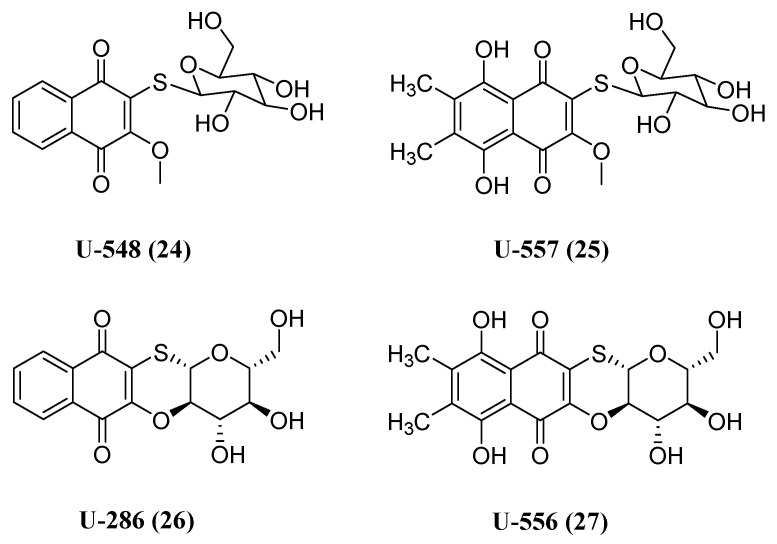
Chemical structure of 1,4-naphthoquinone thioglucosides and their tetracyclic conjugates with inhibitory activity toward P2X7 receptor.

Besides the neurotoxic impact of aggregated proteins, neuroinflammation stands out as a primary contributor to neurodegenerative diseases, including Alzheimer’s disease (AD) and PD [94]. Upon activation, immune cells located within the central nervous system, such as microglia, generate a cascade of inflammatory mediators that harm neurons. Additionally, the release of extracellular ATP, primarily due to cell death, can trigger purinergic receptors on the surface of glial cells, ultimately prompting the synthesis of neurotoxic cytokines [95]. Among the cellular components involved in the activation of microglia and subsequent pathological changes associated with several neurodegenerative diseases, the P2X7 purinoreceptor plays a pivotal role [96,97]. Given its significance, Pislyagin et al. reported the synthesis of a small library of 1,4-naphthoquinone derivatives and their antagonistic effects against mouse P2X7R. From an initial screening with a Ca^2+^ influx assay, they identified four compounds with promising inhibitory activity (Figure 11). Subsequent biological evaluation of these compounds in Neuro-2a cells evidenced a potent blockade of dye uptake, decrease in ROS and NO production and protection of ATP-mediated toxicity. Molecular docking indicated that the naphthoquinone derivatives might exert their inhibitory activity due to interaction with the allosteric site located in the extracellular region of P2X7R [98].

In conclusion, these reports reinforce the elevated potential of naphthoquinone derivatives as neuroprotectors with varied mechanisms of action. In the next section, we will discuss the recent literature on synthetic strategies and a biological evaluation of naphthoquinone compounds targeting PD.

### 3.2. Naphthoquinones with Activity against PD

At the end of the 20th century, epidemiological evidence emerged that smokers had a lower incidence of PD than non-smokers [99,100]. Then, this profile was associated with a lower activity of the enzyme monoamine oxidase (MAO) in the brain of smokers [101]. MAO is an important enzyme for metabolizing amines in the body via oxidative deamination [102]. MAO has two isoforms, MAO-A and MAO-B. The MAO-B isoform is predominant in the brain, where it metabolizes dopamine into 3,4-dihydroxyphenylacetic acid and homovanillic acid [103]. This enzyme is involved in the metabolization of neurotransmitters such as norepinephrine, epinephrine, dopamine and serotonin. The loss of dopamine, norepinephrine and serotonin are the basis for the degenerative process that leads to PD, and there is an association between elevated levels of MAO-B and this neurodegenerative disease [104]. MAO-B inhibition prevents the breakdown of dopamine molecules, which makes them more available to act on their receptors. Its inhibition also decreases the formation of free radicals from dopamine oxidation [105]. Thus, supplementation with L-DOPA and MAO inhibitors is one of the main ways to treat the disease [102].

**Scheme 1:**
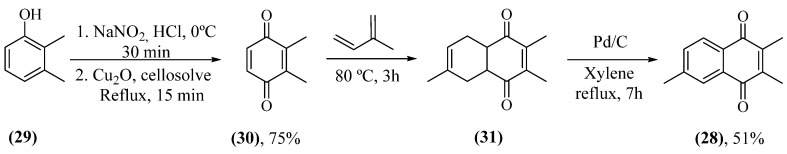
Synthetic route for production of **28**.

With the desire to determine which substances were responsible for the MAO inhibitory activity, a screening of the substances present in burley tobacco leaves was carried out [106]. In this screening, naphthoquinone 2,3,6-trimethyl-1,4-naphthoquinone (**28**) was identified as the main substance responsible for MAO inhibition. Its inhibition was observed as reversible competitive, both against MAO-A and MAO-B, with a K_i_ of 3 μM and 6 μM, respectively. Naphthoquinone **28** was also synthesized for structure confirmation and to provide greater mass for biological assays. The synthesis of this naphthoquinone started with the oxidation of 2,3-dimethylphenol (**29**), with sodium nitrite forming an oxime, followed by an oxidation with copper oxide I producing 2,3-dimethylbenzoquinone (**30**) [107]. Then, **30** reacted by cycloaddition with isoprene, forming the Diels–Alder adduct **31**, which was not isolated. Finally, **31** underwent dehydrogenation, forming **28** (Scheme 1).

Then, **28** was tested in C57BL/6 mice after neurotoxic MPTP treatment [108]. MAO is also the enzyme responsible for converting 1-methyl-4-phenyl-1,2,3,6-tetrahydropyridine (MPTP) to the neurotoxic metabolite, 1-methyl-4-phenylpyridinium ion (MPP+) [109]. Mice treated with **28** and MPTP exhibited a 50% increase in dopamine concentration in the striatum when compared to mice treated with MPTP alone. Thus, under experimental conditions, **28** exerted a neuroprotective effect on mice.

**Figure 12:**
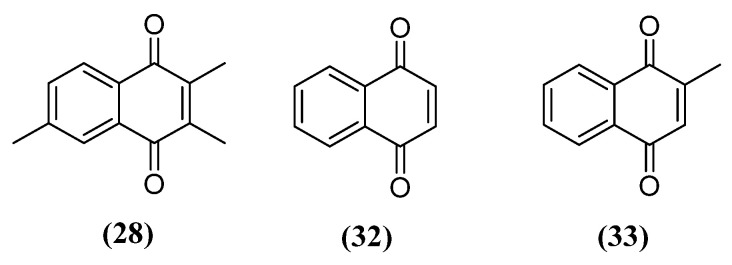
Structure of naphthoquinones with MAO inhibitory activity initially studied.

These works concluded that the naphthoquinone nucleus could be an important pharmacophore for MAO inhibition, which led to the beginning of studies investigating the molecular details of the mechanism of inhibition of 1,4-naphthoquinones. It was discovered that 1,4-naphthoquinone (**32**) and menadione (**33**) (Figure 12) showed strong inhibition of MAO-B, with a K_i_ of 1.4 μM and 0.4 μM, respectively [110]. Regarding MAO-A, a K_i_ of 7.7 μM and 26 μM was found for **32** and **33**, respectively, showing a lower susceptibility of MAO-A in relation to MAO-B with these substances. Both naphthoquinones showed characteristics of reversible and competitive inhibitors for MAO-B. Docking studies suggest that phenyl group interactions at amino acid residues Tyr407 and Tyr444 for MAO-A or Tyr398 and Tyr435 for MAO-B are important for the interaction of the naphthoquinone core and the active site of the enzyme.

**Figure 13:**
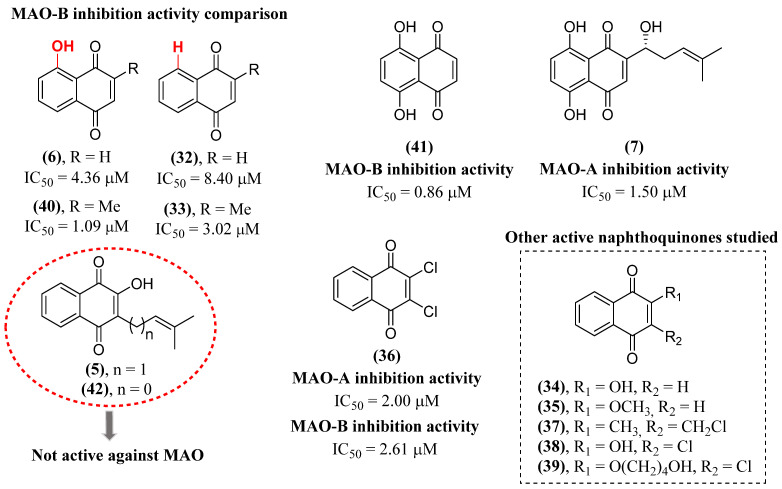
Structure of naphthoquinones studied by Mostert et al. [111].

Continuing with the exploration of this activity of naphthoquinones and contributing to expanding knowledge about the structure–activity relationship, the inhibitory capacity of fourteen natural and synthetic naphthoquinones was studied [111]. In this study (Figure 13), naphthoquinones were grouped according to the degree of hydroxylation of the aromatic ring into 1,4-naphthoquinone analogs (**32**–**39**), juglone analogs (**6**, **40**) and naphthazarine analogs (**41**, **7**), in addition to lapachol (**5**) and *nor*-lapachol (**42**) (Figure 13). Naphthoquinones naphthazarine (**41**) (IC_50_ = 0.860 ± 0.241 μM) and shikonin (**7**) (IC_50_ = 1.50 ± 0.302 μM) showed the greatest capacity to inhibit MAO-B and MAO-A, respectively. Similar results were also found previously when identifying shikonin (**7**) as one of the main substances responsible for the MAO inhibitory effect in *Lithospermum erythrorhizon* extract [112]. An interesting aspect observed was that all naphthoquinones with a hydroxyl in the aromatic ring showed a higher MAO-B inhibitory activity than their non-hydroxylated analogs, as in the example of the comparison of **32** (IC_50_ = 8.40 ± 1.20 μM) with **6** (IC_50_ = 4.36 ± 0.180 μM) and **33** (IC_50_ = 3.02 ± 0.638 μM) with **40** (IC_50_ = 1.09 ± 0.063 μM). Therefore, the presence of hydroxyls in the aromatic ring in the *peri* position in relation to carbonyls may be an important structural aspect for the development of naphthoquinones with MAO inhibitory activity. However, the presence of a C2 hydroxyl does not help this inhibitory activity since, while **32** is an MAO inhibitor, its C2 hydroxylated analog, **34**, showed no activity. Interestingly, **34** and its tautomers extensively perform hydrogen bonding and π stacking according to docking experiments and therefore an explanation for this observation cannot yet be realized. It was also possible to note that the presence of a methyl in C2 is associated with an inversion of selectivity. The C2-methylated analogs tended to be more selective for MAO-B, while the demethylated ones tended to be more selective for MAO-A. 2,3-dichloro-1,4-naphthoquinone (**36**) was also highlighted for its potency against MAO-A (IC_50_ = 2.00 ± 0.878 μM) and MAO-B (IC_50_ = 2.61 ± 0.616 μM). Naphthoquinones **5** and **42** were not identified as MAO inhibitors. 

α-Synuclein is a 14 kDa cytosolic protein composed of 140 amino acids and divided into an N-terminal amphipathic α-helix, a non-amyloid component and a C-terminal acidic tail [113]. This protein presents its aggregation and accumulation as one of the hallmarks of PD [114]. This accumulation produces proteinaceous inclusions known as Lewy bodies or Lewy neurites [115]. α-Synuclein aggregates cause damage and destabilize several processes within the neuronal cell, such as interrupting the formation of the synaptic vesicle release complex, its mobility and dopamine release [116]. Regarding its activity in mitochondria, α-synuclein can inhibit complex I of the respiratory chain, increase the formation of reactive oxygen species (ROS) and interfere with the fusion, fission and potential of the mitochondrial membrane [115,117]. Accumulation of α-synuclein in the endoplasmic reticulum (ER), an essential organelle for protein metabolism, interferes with protein folding, causing stress in the ER [118]. With the broad spectrum of important activities in neurodegenerative diseases of naphthoquinones, the activity of vitamin K analogs and 1,4-naphthoquinone itself (**32**) (Figure 12) on α-synuclein fibrillation was investigated. The results showed that these naphthoquinones, in addition to inhibiting MAO, also have the potential to inhibit α-synuclein aggregation [119]. All naphthoquinones demonstrated a dose-dependent inhibition of fibril formation by α-synuclein with EC_50_ between 15 and 30 µM. Even though many substances show inhibition of α-synuclein fibrillation through non-specific hydrophobic interactions, the largest aliphatic chain of phytoquinone (**13**) (EC_50_ = 29 μM) and menaquinone (**14**) (EC_50_ = 30 μM) (Figure 7) in relation to menadione (**33**) (EC_50_ = 18 μM) and **32** (EC_50_ = 15 μM) (Figure 12) did not translate into a better inhibitory activity. Naphthoquinones **32** and **33** were able to destabilize preformed fibrils, which may be associated with their lower molecular volume allowing for greater diffusion within the fibrillar structure.

The tau protein has an important activity in the growth and development of neurons. The aggregation of tau proteins causes the destabilization of microtubules, which leads to severe consequences for the neuron, such as, for example, the disturbance of axonal transport and neurite outgrowth and leaving the DNA prone to suffering damage. These consequences can lead to cell death [120]. The deposition of tau protein aggregates is an important point in several neurodegenerative diseases, such as PD. The naphthoquinone shikonin (**7**) (Figure 2) was able to decrease the rate and extent of tau aggregation [121]. This substance showed an IC_50_ of 1.2 ± 0.06 and 1.0 ± 0.3 μM for the oligomerization and aggregation of tau 4R2N, respectively, in a heparin-containing medium. The IC_50_ for the inhibition of RNA- and arachidonic-acid-induced tau aggregation was 1.0 ± 0.2 and 1.5 ± 0.1 μM, respectively. Therefore, **7** was able to inhibit tau aggregation in a heparin, RNA and arachidonic-acid-containing medium. Naphthoquinone **7** not only decreased tau oligomerization, but also decreased the average size of tau oligomers. Added to this, with a DC_50_ of 6.3 ± 0.4 μM, **7** was able to disaggregate preformed tau filaments. Tau oligomers were added to a SH-SY5Y neuroblastoma cell culture medium pretreated with **7**. The viability of SH-SY5Y cells treated with 8 µM of tau oligomers rose from 34 ± 2 to 71 ± 6% when pretreated with 250 nM of **7**. The IC_50_ of **7** was significantly lower than other known inhibitors. Planar rings of **7** containing delocalized π electrons can interact with aromatic or polar side chains of tau, which can modify the protein’s conformation. This conformational change would make tau less likely to form aggregates.

Naphthoquinone **7** is associated with several other activities that promote neuroprotective effects in PD models. This naphthoquinone is associated with a decrease in ROS levels attributed to an increase in the activity of the antioxidant enzyme glutathione peroxidase 1 (GPX-1) [122]. Naphthoquinone **7**, at a concentration of 10 μM, induced an increased expression of GPx-1 in PC12 cells, increasing cell survival by 70% against 6-hydroxydopamine (6-OHDA) toxicity [123]. Naphthoquinone **7** also increased the expression of the glutathione-independent antioxidant enzyme, superoxide dismutase 2 (SOD-2), increased the expression of Bcl-2 and decreased the expression of Bax, which reduced the rate of nuclear morphology change that normally occurs in apoptosis. This was the first time that **7** was reported to protect dopaminergic neurons.

Pin1 is a peptidyl-prolyl isomerase that has a profound impact on the regulation of cell growth and proliferation, stress response, immune function, neuronal differentiation, cell motility and apoptosis [124]. Pin1 has already been found in Lewy bodies in PD and also facilitates the formation of α-synuclein inclusions in a cellular model of α-synuclein aggregation. It has also been found that Pin1 is expressed in 50–60% of Lewy bodies in PD patients [125]. Thus, Pin1 is a degenerative factor for dopaminergic neurons. Juglone (**6**) (Figure 2) is a naphthoquinone with recognized Pin1 inhibitory activity [126]. Naphthoquinone **13** (Figure 7) irreversibly inhibits the enzymatic activity of Pin1 by modifying the thiol groups of cysteine residues. Previously, it had already been shown that **6** at a concentration of 5.7 μM in vitro blocks the activity of Pin1 without impacting the activity of other isomerases [127]. Treatment with **6** attenuates Pin1 expression, protects the nigrostriatal axis and improves hypolocomotion in a preclinical model of rats treated with MPTP at a dose of 3 mg/kg, which is non-toxic [128]. In this work, it was found that **6** at 1 μM concentration attenuates MPP+-induced Pin1 expression in MN9D cells. Substance **6** also restored dopamine reuptake in primary mesencephalic neurons and their neurites from MPP+ toxicity. It was also found that **6** restored the behavioral activities as well as the levels of dopamine and its metabolites in the striatum of rats treated with MPTP. Interestingly, even though juglone has been a recognized Pin1 inhibitor for many years, there is a large gap in the literature as to how structural modifications could affect its inhibitory activity.

Naphthazarine (**41**) (Figure 13) at subtoxic doses was investigated for its ability to protect the brain through the activation of adaptive stress response pathways in an animal model of MPTP-induced PD [129]. In this work, **41** at 1 mg/kg significantly prevented the decrease in motor function caused by MPTP. Pretreatment with **41** effectively reduced the loss of dopaminergic neurons in the substantia nigra and loss of nerve endings in the striatum. It was identified that **41** also decreased the activation of astrocytes and microglia in the MPTP model by observing a decrease in GFAP and Iba-1 markers.

**Figure 14:**
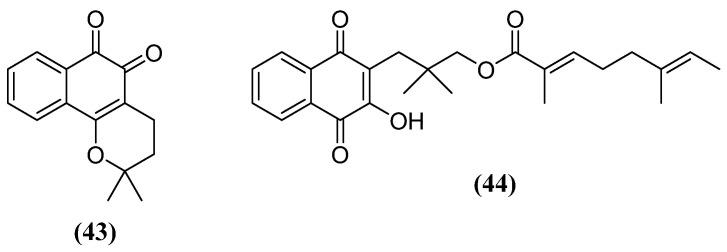
Other natural naphthoquinones with neuroprotective activity for PD.

It has also been found that β-lapachone (**43**) (Figure 14) promotes a neuroprotective effect in rats in a PD model with the administration of MPTP [130]. Naphthoquinone **43** improved impaired movement in MPTP-administered rats through the recovery of dopaminergic neurons in the substantia nigra and striatum and expression of Bcl-2. In this study, it was found that **43** increased the expression of the enzyme heme oxygenase-1 (HO-1), which has antioxidant activity, and of the p-AMPK and Nrf2 signaling molecules in astrocytes of MPTP administered rats. In a study of the co-treatment of **43** with L-DOPA, **43** alleviated dyskinesia induced by chronic L-DOPA treatment in a 6-OHDA-induced mouse model of PD [131]. Dyskinesia is a serious motor problem that occurs in about half of patients when L-DOPA is given to PD patients for a long period of time. Substance **43** decreased the abnormal involuntary movements. Co-treatment substantially decreased astrocyte activation in the striatum and substantia nigra in a unilateral 6-OHDA model.

It was verified that the treatment of rats with 20 mg/kg of rinacanthine C (**44**) (Figure 14) significantly decreased the cataleptic effect of haloperidol when compared to control groups [132]. Treatment with **44** also significantly increased levels of dopamine, norepinephrine and serotonin when compared to the diseased control group. The treatment also increased the rats’ exploratory behavior, which had declined due to the effects of the disease. Improvement in these non-motor symptoms is associated with the restoration of serotonin and dopamine levels in the rat brains. Treatment with **44** normalized brain antioxidant losses at all doses tested. No significant side effects were found in other organs of the body, which could mean that it is a safer profile that should be investigated.

**Scheme 2:**
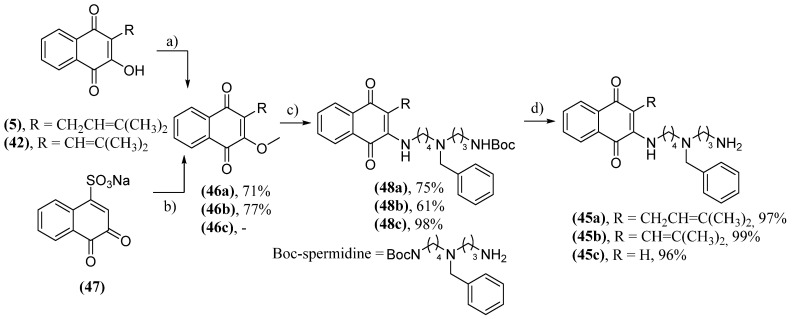
Synthesis of spermidine-conjugated naphthoquinones **45**; (**a**) (CH_3_O)_2_SO_2_, acetone, K_2_CO_3_; (**b**) MeOH, H_2_SO_4_, D; (**c**) MeOH, 90 °C, Boc-spermidine; (**d**) TFA/CH_2_Cl_2_, MeOH, 0 °C.

Considering previous works that showed that naphthoquinones have the capacity to inhibit MAO, the conjugation of these substances with substrate groups for the enzyme could help in the inhibitory activity. Therefore, spermidine-conjugated naphthoquinones (**45**) derived from lapachol (**5**), lawsone (**34**) and *nor*-lapachol (**42**) were synthesized (Scheme 2) [133]. For the production of these molecules, initially, the hydroxyl of the quinonoid ring was methylated. The methylated derivatives of **5** and **42**, **46a** and **46b**, respectively, were produced by reacting these naphthoquinones with dimethylsulfate in acetone and potassium carbonate [134]. The methylated derivative of **34**, **46c**, was synthesized from the sodium salt of 1,2-naphthoquinone-4-sulfonic acid (**47**) [135]. Next, the methoxy group underwent a nucleophilic displacement by the Boc-protected spermidine, producing **48**. The naphthoquinones **45** were then obtained with the removal of the protection group (Scheme 2). Naphthoquinones **45** showed a K_i_ between 12 and 63 μM with low selectivity between MAO-A and MAO-B and a kinetic study compatible with a competitive mechanism. Although **34** had the lowest K_i_ compared to the other naphthoquinones, all other derivatives with substituents on the quinonoid ring were less potent than 1,4-naphthoquinone (**32**). All these naphthoquinones showed characteristics of reversible inhibitors. The greater activity of **42** compared to **5** may be associated with its closer proximity to the FAD portion as well as the orientation of the quinonoid ring in the aromatic stacking of Tyr435/Tyr398 residues. Lapachol (**5**) showed no inhibitory activity, while *nor*-lapachol (**34**) showed low activity against MAO-A, which may be due to the absence of hydrogen bonds between naphthoquinone and the catalytic site, as previously reported [89]. Using the same reasoning, **45c** was the naphthoquinone–spermidine with the highest potency for MAO-B inhibition, which may be associated with the greater number of hydrogen bonds between naphthoquinone and the FAD moiety at the catalytic site.

**Scheme 3:**
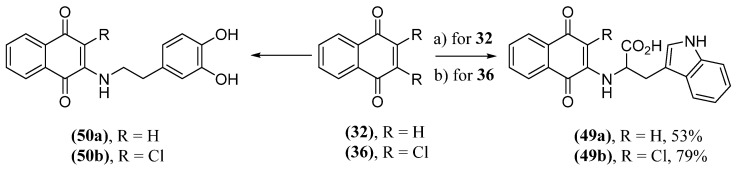
Synthesis of hybrids of naphthoquinones with tryptophan (**49**) and dopamine (**50**); (**a**) L-tryptophan, EtOH/H_2_O, 23 °C, 24 h; (**b**) L-tryptophan, KOH, MeOH/H_2_O, 24 h.

There are works in the literature that demonstrate the ability of hybrids of naphthoquinones and tryptophan (**49**) (Scheme 3) to inhibit the aggregation of the amyloid-beta peptide [136,137]. These naphthoquinones were also tested as potential inhibitors of α-synuclein protein aggregation [138]. In the same context, dopamine can form adducts with α-synuclein in vitro, inhibiting the formation of fibrils. This dopamine activity, along with the already known ability of naphthoquinones to inhibit α-synuclein aggregation, stimulated the synthesis of dopamine and naphthoquinone hybrids (**50**) (Scheme 3) [139]. These naphthoquinones were produced by means of a Michael addition to the quinonoid ring by tryptophan or dopamine followed by replacement of the hydrogen or chlorine atom to produce **49a** and **49b** or **50a** and **50b**, respectively (Scheme 3) [140]. The two naphthoquinones **49** demonstrated a dose-dependent inhibition of fibril formation by α-synuclein. Analog **49a** showed a better inhibition capacity, which may be associated with lower steric hindrance. The synthesis of hybrids **50** occurred similarly to the production of hybrids **49**; however, the reaction conditions and yields were not mentioned. Maximum inhibition was observed with a hybrid concentration five times higher than α-synuclein by the ThT assay, being 88% for **50a** and 76% for **50b**. Analysis of data from experiments carried out with **32** and dopamine demonstrate a superior inhibitory activity of **50a** and that the presence of the naphthoquinone structure and dopamine in the same molecule has a synergistic effect.

**Scheme 4:**
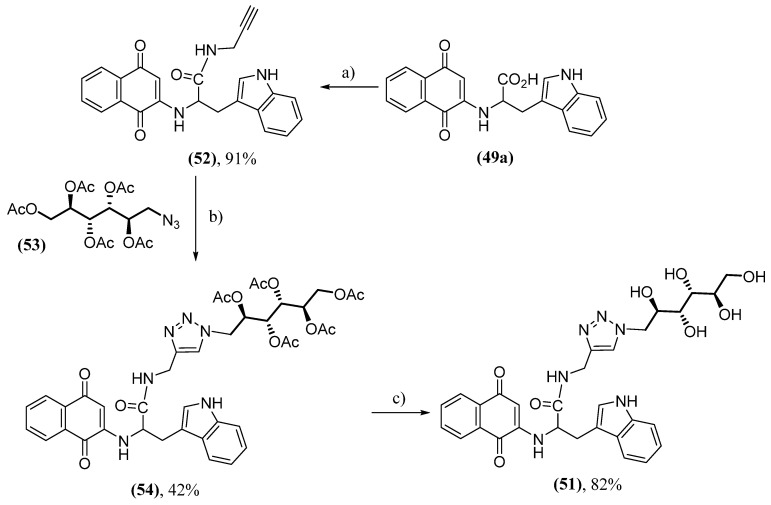
Synthesis of naphthoquinone conjugated with mannitol **51** by click reaction; (**a**) propargylamine, EDCI, HOBt, DIEA, THF/DCM; (**b**) **53**, sodium ascorbate, CuSO_4_; (**c**) CH_3_ONa/CH_3_OH, pH = 8.5.

Hypothesizing that conjugating these naphthoquinones with mannitol could enhance their ability to access the blood–brain barrier, analogs of naphthoquinone **49a** conjugated with mannitol were synthesized [141]. These naphthoquinones were synthesized by click reactions (**51**) or PEG connector (**52**). These molecules have been shown to reduce the kinetics and extent of α-synuclein aggregation. Furthermore, the conjugates are more potent than **49a** or its mixture with mannitol. The synthetic route to producing naphthoquinone **51** starts from **49a** with an amide formation reaction with propargylamine, producing **52**. In the terminal alkyne group of **52**, there is a click reaction with the azide **53** to form the triazole (**54**), ending with hydrolysis of the acetate groups (Scheme 4).

**Scheme 5:**
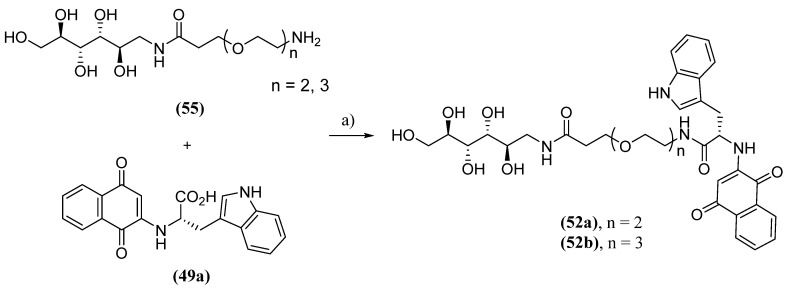
Synthesis of naphthoquinones conjugated with mannitol (**52**) by PEG connector; (**a**) **55**, DIEA, HOBT, EDC.Cl, DMSO, r.t., 12 h.

Naphthoquinone **52b** was produced by amide group formation from the PEG connector **55** with the carboxyl of **49a** (Scheme 5) and showed the best inhibitory activity. It is speculated that the longer PEG chain in **52b** may confer greater flexibility and, consequently, a more efficient interaction with α-synuclein.

**Scheme 6:**
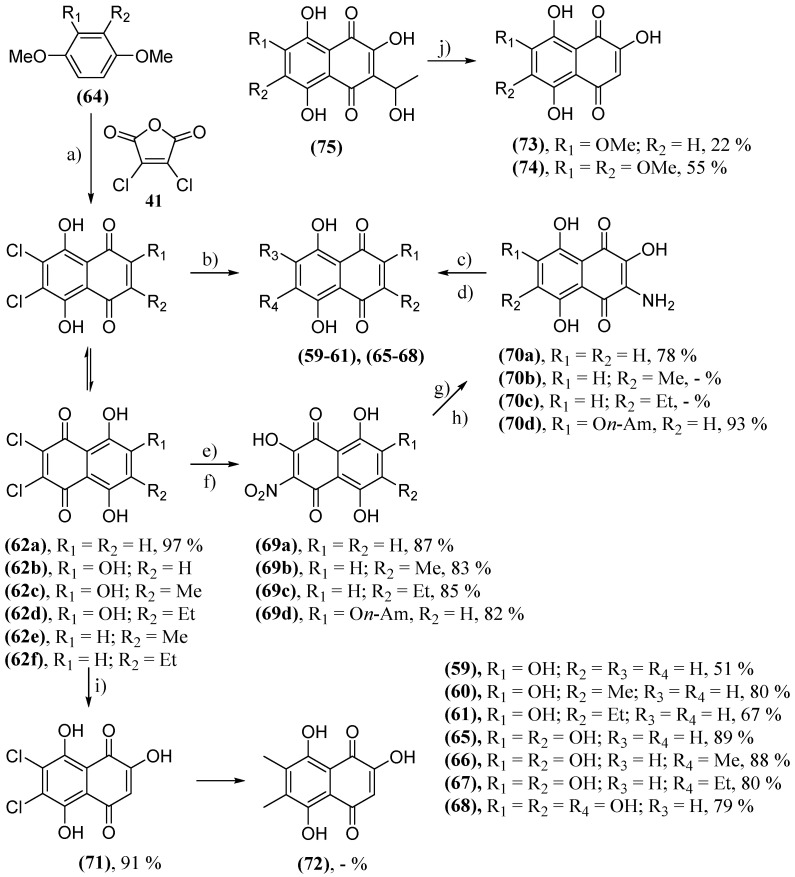
Synthesis of naphthazarine (**41**) analogs. (**a**) AlCl_3_, NaCl, 190 °C; (**b**) for **62b–d**: 1. Fe, HOAc; 2. [O]; (**c**) for **70a–c**: DMSO, HCO_2_H, H_2_SO_4_, H_2_O, reflux; (**d**) for **70d**: 1. DMSO, HCO_2_H, H_2_SO_4_, H_2_O, reflux; 2. MeSO_3_H, HCO_2_H, reflux; (**e**) for **62a,e,f**: 1. NaNO_2_, acetone-MeOH, reflux, 2 h; 2. HCl c.; (**f**) for **62b**: 1. isoamyl alcohol, H_2_SO_4_, reflux; 2. NaNO_2_, acetone-MeOH, reflux, 2 h; 3. HCl c.; (**g**) for **69a–b**: Na_2_S_2_O_4_, H_2_O, 2 h; (**h**) for **69d**: Na_2_S, H_2_O, r.t.; (**i**) for **62a**: MnO_2_, H_2_SO_4_, r.t., 2 h; (**j**) NaOH/H_2_O, reflux.

Considering the already explored protective effect of naphthazarine and other structurally analogous naphthoquinones, Aminin and collaborators evaluated the neuroprotective activity in Neuro-2a neuroblastoma cells in a model of PD of numerous natural naphthoquinones and their synthetic derivative. The tested naphthoquinones were divided into four groups: naphthazarin (**41**) and its analogs; derivatives with acetylated *O*-glucoside groups (**56**); derivatives with acetylated *S*-glucoside groups (**57**); derivatives with deacetylated *S*-glucoside groups (**58**). The analogs of **41** were obtained by various methods. Naphthopurpurine (**59**), 2-hydroxy-3-methylnaphthazarine (**60**) and 2-hydroxy-3-ethylnaphthazarine (**61**) were obtained by a reductive dechlorination of **62** using iron in acetic acid followed by air oxidation [142]. One way to obtain the dichlorinated derivatives **62** is through the Friedel–Crafts cycloaddition between a hydroquinone and dichloromaleic anhydride (**63**), as in the case of the synthesis of **62a**, which uses hydroquinone **64** [143]. 2,3-Dihydroxynaphthazarine (**65**), 2,3-dihydroxy-6-methylnaphthazarine (**66**), 2,3-dihydroxy-6-ethylnaphthazarine (**67**) and spinochrome D (**68**) were synthesized using an efficient synthetic route starting from **64**. Naphthoquinone **64** reacted with sodium nitrite, forming intermediate **69**, followed by a reduction producing 3-amino-2-hydroxynaphthazarines (**70**) [144]. Naphthoquinones **70** then underwent dimethylsulfoxide-mediated oxidation [145,146]. 2-hydroxy-6,7-dimethylnaphthazarine (**71**) was prepared by oxidation **64a** with MnO_2_ in concentrated acid, while 6,7-dichloro-2-hydroxy-naphthazarine (**72**) was produced by a nucleophilic substitution of chlorine atoms by methyl groups, but the reaction conditions were not described [147]. Finally, 2-hydroxy-7-methoxynaphthazarine (**73**) and 2-hydroxy-6,7-dimethoxynaphthazarine (**74**) were synthesized by a retro aldol disintegration from their 2-hydroxy-3-(1-hydroxy-ethyl) corresponding naphthazarins (**75**) (Scheme 6) [148].

**Scheme 7:**
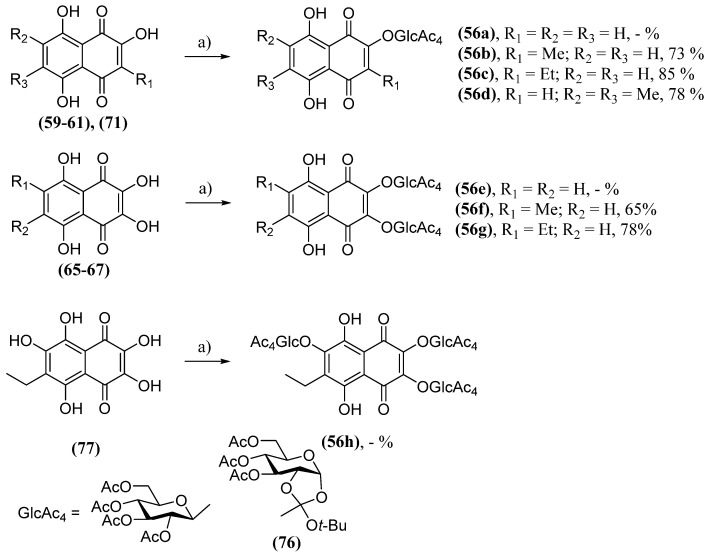
Synthesis of acetylated triglucosidyl derivatives (**56**); (**a**) **76**, chlorobenzene, reflux.

These naphthoquinones were converted into their derivatives with acetylated *O*-glucoside groups (**56**) by means of a condensation with 3,4,6-tri-*O*-acetyl-α-d-glucopyranose 1,2-(tert-butoxy orthoacetate) (**76**) [149,150]. The amount used was equimolar to the number of β-hydroxy groups. Thus, the glycosidation of 2-hydroxy-naphthazarines (**59**–**61**, **71**), 2,3-dihydroxy-naphthazarines (**65**–**67**) and echinochrome A (**77**) formed the derivatives mono- (**56a**–**d**), bis- (**56e**–**g**) and acetylated tris-*O*-glycosides (**56h**), respectively (Scheme 7). The glycosylation of these naphthoquinones was carried out in order to improve the solubility of these molecules and achieve a targeted action.

**Scheme 8:**
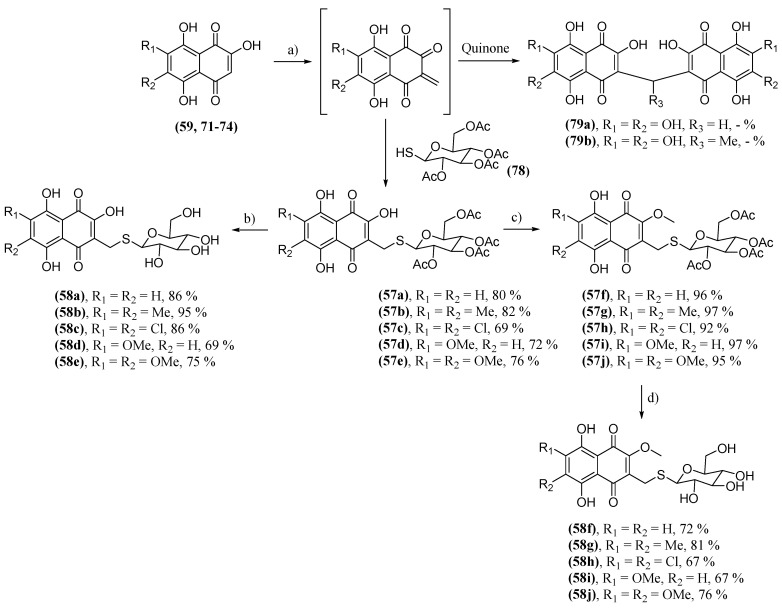
Synthesis of thioglucosidyl derivatives **57** and **58**; (**a**) **78**, paraformaldehyde, HCOOH, acetone, reflux; (**b**) HCl/MeOH; (**c**) 1. CH_2_N_2_; 2. HCl/MeOH; (**d**) HCl/MeOH.

It was found that the deacetylation of derivatives **56** produced very unstable molecules. This led to the need for the synthesis of thioglycosylated derivatives with the moiety of **41** [150]. These thioderivatives were synthesized due to the known stability of thioglycosides to acid and basic hydrolysis, and they did not undergo enzymatic degradation. The 2-hydroxynaphthazarines (**59**, **71**–**74**) were used for the synthesis of derivatives with acetylated *S*-glucoside groups (**35**). Tetra-*O*-acetyl-1-mercapto-D-glucose (**78**) was used as a thiol source. The reaction starts with a Knoevenagel condensation between the naphthazarine analog and paraformaldehyde, forming methide *o*-quinone. Next, there is a nucleophilic attack of **77**, forming conjugates **57**. Another possibility is that the *o*-quinone methide reacts with another molecule of the naphthazarine analog, forming dimers. Two produced dimers were included in the biological tests, **79a–b**, which are spinochrome D (**68**) dimers linked by a methylene or ethylidene bridge. Conjugates **57** were then deacetylated in acid solution, forming derivatives with deacetylated *S*-glucoside groups (**58**), or they were methylated at the 2-position hydroxyl with a diazomethane solution, yielding **57f–j**. These also underwent subsequent deacetylation, yielding **58f–j** (Scheme 8).

Most naphthoquinones tested were able to protect Neuro-2a cells against the neurodegenerative effects of neurotoxins. Naphthoquinone **74** exhibited the greatest neuroprotective effect in the paraquat model during the MTT test, increasing the number of viable cells by 45.7% at a concentration of 0.1 μM compared to the control. The neuroprotective effect was greater in the paraquat model than in the 6-OHDA model. In this model, spinochrome D (**68**) had the best result, with an increase of only 22.3% of viable cells compared to the control. Naphthoquinones **65**, **66**, **74**, **79b** and **58h** had the best results in improving cell survival in MTT, non-specific esterase and PI fluorescent dye tests, both in paraquat and 6-OHDA models. Naphthoquinone **65** was also the substance with the greatest ability to decrease ROS production in the paraquat and 6-OHDA models, decreasing production by 30.2% (0.1 μM) and 31.6% (1.0 μM), respectively. Incubation with paraquat resulted in a charge shift in the mitochondrial membrane potential (MMP), of which, **65** and **66**, at the 0.01 µM concentration, were able to increase by 8.9% and 21.2%, respectively. Of all the tests, **74** was the most active in terms of its neuroprotective capacity, while naphthazarine (**41**) was the most inactive. The QSAR study suggests that the greater activity of **74** is related to a greater number of hydrogen bond acceptor sites and greater hydrophobic surface. Naphthoquinone **41**, on the other hand, showed a greater number of hydrogen bond donor sites and a significantly smaller hydrophobic region. These naphthoquinones demonstrated the protection of non-specific esterase activity from the inhibitory activity of neurotoxins, normalized the cell cycle and inhibited the lytic activity of membranes. These activities are related to the ability of these naphthoquinones to decrease ROS production. For naphthoquinone **55**, β-hydroxyl groups are essential for the antioxidant effect, which explains the lower neuroprotective activity in methoxylated derivatives [151]. Glycosidation with *O*- and *S*-glycoside groups, acetylated or not, blocked β-hydroxyl groups and were responsible for the low neuroprotective activity of these derivatives.

The acetylated triglucosidic derivative of echinochrome A (**56h**) was used to activate the transcription factor HSF1 and increase the expression of the molecular chaperone HSP70 [152]. This naphthoquinone activity is already known, and it was used in this work to provide more evidence that the elevation of HSP70 levels is a possible therapeutic target for the treatment of PD. This activity reverses the neurodegeneration process, as HSP70 has the ability to prevent α-synuclein aggregation and microglia activation. Naphthoquinone **56h** was also indicated as therapeutically effective since, in an animal model with lactacystin-induced PD, it reduced the loss of dopaminergic neurons by two times when compared to the control, protected the activity of neurons in the substantia nigra pars compacta, eliminated motor dysfunction characteristic of PD, reduced microglial activation and decreased neuroinflammation. When tested in aged rats in a lactacystin-induced preclinical PD model, the **56h** administration led to a 130% increase in HSP70 compared to the control, which decreased neurodegeneration by 2.4 times [153].

As discussed throughout the text, naphthoquinones exert biological activity on many targets of interest in the development of active molecules against PD. Naturally, different therapeutic targets present different interactions with the molecular framework of naphthoquinones to achieve the desired performance. Regarding MAO inhibition, naphthoquinones that presented hydroxyls on the aromatic ring in the *peri* position in relation to the carbonyl showed greater inhibitory activity. In fact, it is worth highlighting that in many studies presented in this review, naphthoquinones hydroxylated or polyhydroxylated on the aromatic ring had the best activity against their respective biological targets. Researchers who intend to develop new molecules to act on these targets must take these structural aspects into account when planning new molecules. Among natural naphthoquinones, the inhibition of α-synuclein aggregation was more successful with naphthoquinones with a smaller molecular volume and short aliphatic chains associated with the quinonoid ring. However, it should be noted that, in most studies, there was not a wide variety of molecules being tested, which gives studies on structure–activity relationships a more speculative tone. An example of this is that, among the synthetic naphthoquinones, **52b** presented the best inhibitory activity for α-synuclein aggregation even though it has the largest PEG chain associated with the quinonoid ring.

There are still many structural modifications that can be explored in the development of bioactive molecules against PD. Although β-lapachone (**43**) presents neuroprotective activity in rats in the PD model, there are still very few studies on the behavior of the activity of 1,2-naphthoquinones in targets that can act against this disease. Juglone (**6**) is already recognized as a Pin1 inhibitor molecule. With its reactive electrophilic sites on the quinonoid ring, **6** can be used to produce a wide range of new structures and possibly new Pin1 inhibitory molecules.

## 4. Conclusions

In this review, we describe the worldwide epidemiology of PD, including its risk factors, clinical manifestations and pathophysiological mechanisms. As oxidative stress is a pathological manifestation of traumatic brain injuries such as PD, the use of antioxidant drugs, such as those belonging to the 1,4-naphthoquinone class, may be an effective alternative in the treatment of this disease. For this reason, we report many examples of 1,4-naphthoquinones that have shown neuroprotective effects. In addition, we also highlight the main biological targets related to the neurological disorders that cause PD and the naphthoquinones that showed promising results in assays directed toward these targets. The synthetic routes chosen for the production of new naphthoquinone derivatives were also highlighted in this review. 

Despite the promising results presented, there are still challenges to be overcome in the use of naphthoquinones in the treatment of PD. It is necessary to investigate the action of these compounds in clinical trials in humans, aiming to assess whether the effects observed in cellular and animal models can also be verified in clinical practice. Furthermore, other studies need to be carried out to better understand the interaction of the various promising compounds with the therapeutic targets of the disease. This review provides a survey of the literature on 1,4-naphthoquinones that can be explored to fill these gaps.

## Data Availability

Data sharing is not applicable.
